# Supplementary material for: Exogenous Melatonin Reprograms the Rhizosphere Microbial Community to Modulate the Responses of Barley to Drought Stress
Source: Int J Mol Sci. 2022 Aug 26;23(17):9665. doi: 10.3390/ijms23179665 (PMC9456345; doi:10.3390/ijms23179665)
Supplement: Supplementary file 1 [file ijms-23-09665-s001.zip › ijms-1836869-supplementary.pdf]

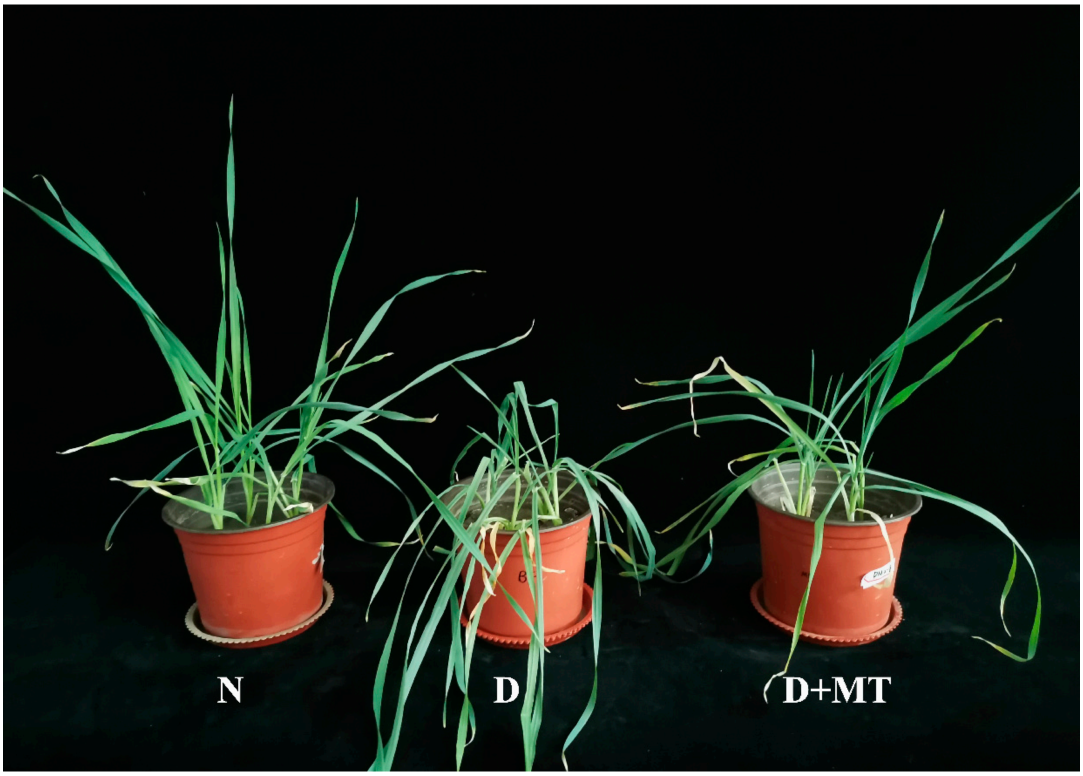

Figure S1. Morphology of barely as affected by melatonin and drought stress. N, normal control; D, drought stress; D+MT, melatonin + drought stress.

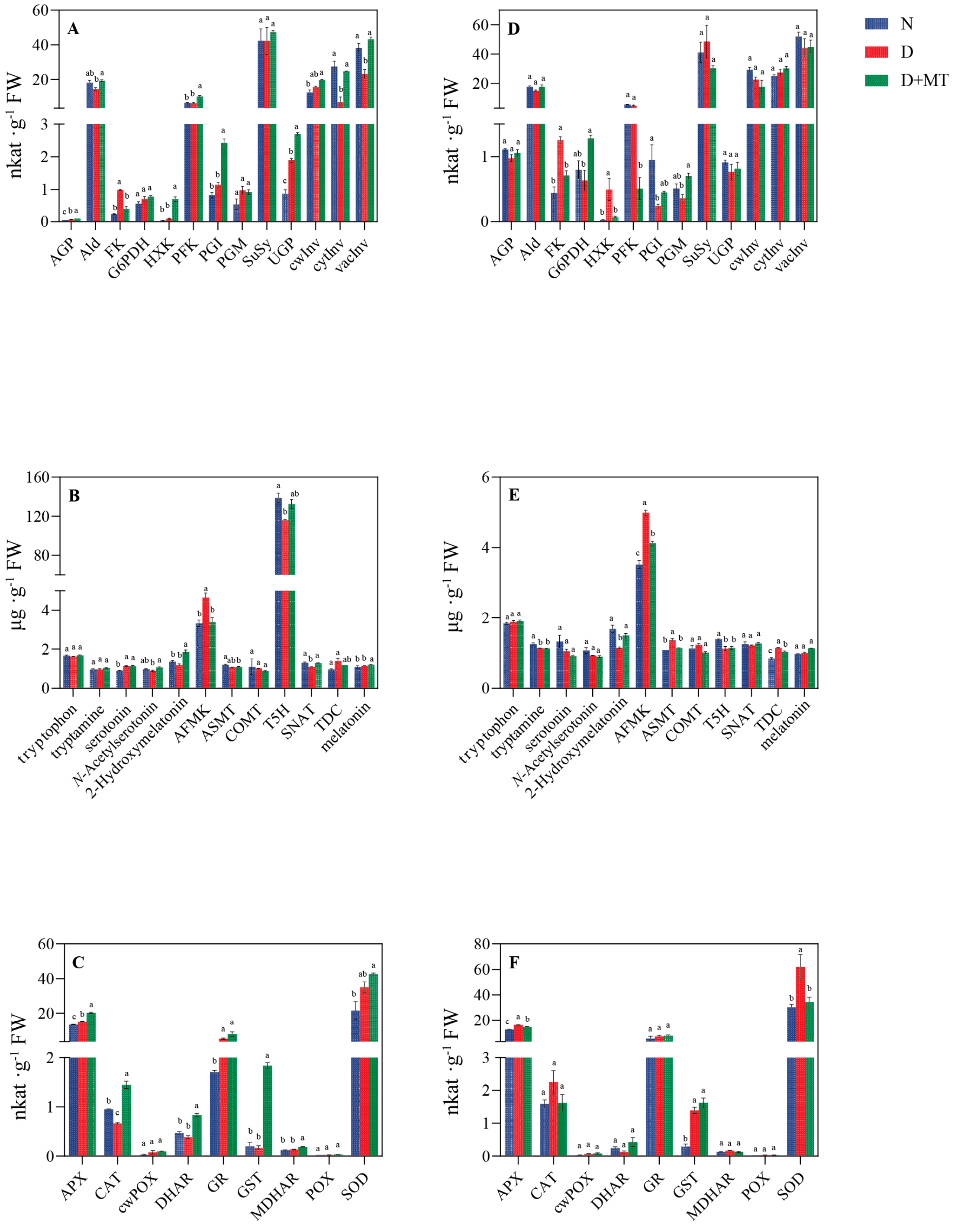

Figure S2 Metabolite concentrations and activities of enzymes involved in metabolisms of reactive oxygen species, carbohydrate, and melatonin in barely root (A, B, C) and leaf (D, E, F) as affected by melatonin treatment and drought stress. SOD, superoxide dismutase; cwPOX, cell wall peroxidase; CAT, catalase; POX, peroxidase; APX, ascorbate peroxidase; MDHAR, monodehydroascorbate

reductase; DHAR, monodehydroascorbate; GR, glutathione reductase; GST, glutathione S-transferase; cytInv, cytoplasmic invertase; vacInv, vacuolar invertase; cwInv, cell wall invertase; UGPase, UDP-glucose pyrophosphorylase; Susy, sucrose synthase; HXK, hexokinase; G6PDH, glucose-6-phosphate dehydrogenase; PGI, phosphoglucoisomerase; FK, fructokinase; PFK, phosphofructokinase; PGM, phosphoglucomutase; AGPase, ADP-glucose pyrophosphorylase; Ald, aldolase; T5H, Tryptamine 5-hydroxylase; AFMK, N^1-acetyl-N^2-formyl-5-methoxyknuramine; TDC, Tryptophan decarboxylase; SNAT, Serotonin *N*-acetyltransferase; ASMT, *N*-acetylserotonin methyltransferase, COMT, caffeic acid, *O*-methyltransferase; MT, melatonin. Different small letters represent significant differences at $P < 0.05$. Data are expressed as means ± SEM (n=4).
